# Lipidomic Analyses Reveal Specific Alterations of Phosphatidylcholine in Dystrophic *Mdx* Muscle

**DOI:** 10.3389/fphys.2021.698166

**Published:** 2022-01-12

**Authors:** William J. Valentine, Sherif A. Mostafa, Suzumi M. Tokuoka, Fumie Hamano, Natsuko F. Inagaki, Joel Z. Nordin, Norio Motohashi, Yoshihiro Kita, Yoshitsugu Aoki, Takao Shimizu, Hideo Shindou

**Affiliations:** ^1^Department of Molecular Therapy, National Center for Neurology and Psychiatry (NCNP), National Institute of Neuroscience, Kodaira, Tokyo, Japan; ^2^Department of Lipid Signaling, National Center for Global Health and Medicine (NCGM), Shinjuku-ku, Japan; ^3^Weill Cornell Medicine—Qatar, Doha, Qatar; ^4^Department of Lipidomics, Graduate School of Medicine, The University of Tokyo, Bunkyo-ku, Japan; ^5^Life Sciences Core Facility, Graduate School of Medicine, The University of Tokyo, Bunkyo-ku, Japan; ^6^Department of Laboratory Medicine, Centre for Biomolecular and Cellular Medicine, Karolinska Institutet, Huddinge, Sweden; ^7^Department of Medical Lipid Science, Graduate School of Medicine, The University of Tokyo, Bunkyo-ku, Japan

**Keywords:** *mdx*, Duchenne, phospholipid, phosphatidylcholine, oleic acid, muscular dystrophy, skeletal muscle

## Abstract

In Duchenne muscular dystrophy (DMD), lack of dystrophin increases the permeability of myofiber plasma membranes to ions and larger macromolecules, disrupting calcium signaling and leading to progressive muscle wasting. Although the biological origin and meaning are unclear, alterations of phosphatidylcholine (PC) are reported in affected skeletal muscles of patients with DMD that may include higher levels of fatty acid (FA) 18:1 chains and lower levels of FA 18:2 chains, possibly reflected in relatively high levels of PC 34:1 (with 16:0_18:1 chain sets) and low levels of PC 34:2 (with 16:0_18:2 chain sets). Similar PC alterations have been reported to occur in the *mdx* mouse model of DMD. However, altered ratios of PC 34:1 to PC 34:2 have been variably reported, and we also observed that PC 34:2 levels were nearly equally elevated as PC 34:1 in the affected *mdx* muscles. We hypothesized that experimental factors that often varied between studies; including muscle types sampled, mouse ages, and mouse diets; may strongly impact the PC alterations detected in dystrophic muscle of *mdx* mice, especially the PC 34:1 to PC 34:2 ratios. In order to test our hypothesis, we performed comprehensive lipidomic analyses of PC and phosphatidylethanolamine (PE) in several muscles (extensor digitorum longus, gastrocnemius, and soleus) and determined the *mdx*-specific alterations. The alterations in PC 34:1 and PC 34:2 were closely monitored from the neonate period to the adult, and also in mice raised on several diets that varied in their fats. PC 34:1 was naturally high in neonate’s muscle and decreased until age ∼3-weeks (disease onset age), and thereafter remained low in WT muscles but was higher in regenerated *mdx* muscles. Among the muscle types, soleus showed a distinctive phospholipid pattern with early and diminished *mdx* alterations. Diet was a major factor to impact PC 34:1/PC 34:2 ratios because *mdx*-specific alterations of PC 34:2 but not PC 34:1 were strictly dependent on diet. Our study identifies high PC 34:1 as a consistent biochemical feature of regenerated *mdx*-muscle and indicates nutritional approaches are also effective to modify the phospholipid compositions.

## Introduction

Phosphatidylcholine (PC) is a main component of the lipid bilayer in plasma cell membranes that surrounds muscle fibers. This bilayer forms a hydrophobic barrier that functions to prevent the unregulated passage of ions and other charged molecules, which would otherwise lead to myofiber death and muscle wasting. Duchenne muscular dystrophy (DMD) is a progressive wasting muscle disease characterized by fragile plasma membranes due to a lack of the structural protein dystrophin in myofibers. An unexplained lipid alteration has been observed for over 50 years in muscles of patients with DMD that involves a large increase in fatty acid (FA) 18:1/oleic-acid chains and a relative decrease in FA 18:2/linoleic-acid chains in PC (Takagi et al., 1968; Kunze et al., 1975; Pearce et al., 1981). In more recent studies, a related alteration was suggested to be reflected in higher ratios of PC 34:1 (containing 16:0_18:1 chain sets) to PC 34:2 (containing 16:0_18:2 chain sets) in the affected muscles (Tahallah et al., 2008; Table 1). In DMD muscle fibers, enhanced susceptibility to contraction-induced membrane stress results in loss of barrier function of the plasma membranes and unregulated influx of calcium, leading to muscle degeneration (Turner et al., 1988; Menke and Jockusch, 1991; Straub et al., 1997). The biological origin and meaning of the altered PC in DMD muscles has been unknown, however, considering the role of acyl chains of phospholipid in biomembrane integrity, the replacement of FA 18:2 chains with FA 18:1 chains is of considerable interest, especially because mono-unsaturated chains like FA 18:1 are more conformationally stable than polyunsaturated chains like FA 18:2 (Feller et al., 2002), and large alterations of the PC 34:1/PC 34:2 ratios may affect biophysical properties of the membranes that impact the disease course.

**TABLE 1:** Phosphatidylcholine alterations reported in the literature.

Subjects	Changes in dystrophic muscle	References
DMD patients	Increased FA 18:1 and decreased FA 18:2 in PC	Takagi et al., 1968
DMD patients	Increased FA 18:1 and decreased FA 18:2 in PC	Kunze et al., 1975
DMD patients	Increased FA 18:1 in PC	Pearce et al., 1981
DMD patients	Increased PC 34:1/PC 34:2 ratio	Tahallah et al., 2008
DMD patients	Increased PC 34:1	Dabaj et al., 2021
*mdx* mice	Increased PC 34:1/PC 34:2 ratio	Touboul et al., 2004
*mdx* mice	Increased PC 34:1/PC 34:2 ratio	Benabdellah et al., 2009
*mdx* mice	Increased FA 18:1 in total phospholipids	Tuazon and Henderson, 2012
*mdx* mice	Increased PC 34:1	Paran et al., 2015
*mdx* mice	PC 34:1 and PC 34:2 both increased (EDL) or unchanged (SOL)	Senoo et al., 2020

The *mdx* mouse, with a stop codon mutation in exon 23 of the dystrophin gene, is a valuable DMD disease model (Hoffman et al., 1987). Despite generally milder course than in human DMD disease, *mdx* mice replicate many DMD-like biochemical and genetic features associated with dystrophin loss including myofiber membrane instability and enhanced permeability which drives progressive muscle wasting (Straub et al., 1997). A similarity in the PC alterations was also suggested by that increased ratios of PC 34:1/PC 34:2 were detected in affected *mdx* mouse muscles (Touboul et al., 2004; Benabdellah et al., 2009) in a similar manner as was detected in DMD patients’ muscles (Tahallah et al., 2008; Table 1). However, other studies indicate that both PC 34:1 and PC 34:2 may be equally elevated in affected *mdx* muscle, in which case the PC 34:1/PC 34:2 ratios would be unchanged (Senoo et al., 2020), which we also observed.

We hypothesized that several experimental factors, including muscle types sampled, mouse ages, and mouse diets, may strongly affect the PC alterations detected in mdx muscles, including the PC 34:1/PC 34:2 ratios. In order to test our hypothesis, we performed lipidomic analyses to detect changes in the abundant membrane phospholipids PC and phosphatidylethanolamine (PE) in several muscles of wild-type (WT) and *mdx* mice during different stages of growth and development, and in mice raised on different diets. Multiple *mdx*-specific alterations were detected in muscles after the initial wave of degeneration at age ∼3 weeks, but with an early and diminished pattern occurring in soleus (SOL) compared to the other muscles sampled extensor digitorum longus (EDL) and gastrocnemius (GAS). Diet was also a critical factor, and high PC 34:1 occurred in *mdx* muscle independently of diets while high PC 34:2 occurred strictly in a diet-dependent manner. Our study identifies high levels of PC 34:1 as a specific biochemical feature of regenerated *mdx* muscles and indicates muscle type age, and diet also strongly impact phospholipid alterations, including PC 34:1/PC 34:2 ratios, in dystrophic muscles.

## Materials and Methods

### Animals Studies

All studies using mice were conducted in accord with the Public Health Service (PHS) Policy on Humane Care and Use of Laboratory Animals. Maintenance of the animal facility and use of animals was in full compliance with the Ethics Committee for animal experiments of the National Center for Global Health and Medicine, Tokyo, Japan.

### Mice

Wild-type C57BL/6N, B10-ScN, and B10-ScSn-Dmd (*mdx*) mice were obtained from CLEA Japan, Inc. (Tokyo, Japan). B10-ScN and *mdx* mice were crossed and female offspring in successive generations heterozygous for the X-linked *mdx* mutation were bred to produce litters containing both B10-wild-type (WT) and -*mdx* male mice, and these male littermates were used for all comparative analyses of WT and *mdx* tissues. The original B10-ScN strain, but not B10-*mdx* strain, harbored a TLR4 deletion mutation which inhibits lipopolysaccharide signaling in a recessive manner (Coutinho and Meo, 1978; Vogel et al., 1979; Du et al., 1999). In the crossing of the strains, the inheritance of the mutated TLR4 allele was monitored and WT and *mdx* mice containing only non-mutant TLR4 alleles were generated, and matched cohorts of heterozygous mice were also used for comparative analyses. All mice were housed in an air-conditioned animal room under specific-pathogen-free (SPF) conditions, with a 12-h light/dark cycle. Mice had *ad libitum* access to food and water and were fed CE-2 standard rodent chow diet (CLEA Japan, Inc., Tokyo, Japan) or custom research diets (described below).

### Genotyping

Genotyping of WT and *mdx* mice was performed from mouse tail genomic DNA extracts. *Mdx* genotyping was performed by primer competition PCR (Shin et al., 2011) using the three-primer set common-forward (5′-GCG CGA AAC TCA TCA AAT ATG CGT GTT AG TGT-3′), WT-reverse (5′-GAT ACG CTG CTT TAA TGC CTT TAG TCA CTC AGA TAG TTG AAG CCA TTT TG-3′), and *mdx*-reverse (5′-CGG CCT GTC ACT CAG ATA GTT GAA GCC ATT TTA-3′) to detect WT (134 base pairs) and *mdx* alleles (117 base pairs). TLR4 genotyping was performed according to the Jackson Laboratory protocol using the primer set mutant forward (5′-GCA AGT TTC TAT ATG CAT TCT C -3′) and mutant reverse (5′-CCT CCA TTT CCA ATA GGT AG-3′) to detect mutant alleles (140 base pairs) and the primer set wild-type forward (5′-ATA TGC ATG ATC AAC ACC ACA G -3′) and wild-type reverse (5′-TTT CCA TTG CTG CCC TAT AG -3′) to detect wild-type alleles (390 base pairs).

### Phospholipid Analysis by LC-MS

Tissue samples (∼5–25 mg) were isolated from the mid-belly region of muscles and stored at −80^°^C. Tissues were pulverized in a frozen state using a Tokken Automill cryogenic pulverizer (Tokken, Japan). After adding methanol to the crushed frozen tissues and incubating at 4°C for 1 h, methanolic extracts of the pulverized samples were collected and centrifuged at 14,000 rpm for 10 min at 4°C. The supernatants were collected, diluted with methanol to a concentration corresponding to ∼3 mg of original tissue/ml, and stored at −80^°^C. For lipidomic analyses, samples were further diluted with methanol ∼20-fold before LC-selected reaction monitoring (SRM)-MS analyses were performed using a Nexera UHPLC system and LCMS-8050 triple quadrupole mass spectrometers (Shimadzu, Japan). An Acquity UPLC BEH C8 column (1.7 μm, 2.1 mm × 100 mm, Waters) was used with the following ternary mobile phase compositions: 5 mM NH_4_HCO_3_/water (mobile phase A), acetonitrile (mobile phase B), and isopropanol (mobile phase C). The pump gradient [time (%A/%B/%C)] was programmed as follows: 0 min (50/45/5)-10 min (20/75/5)-20 min (20/50/30)-27.5 min (5/5/90)-28.5 min (5/5/90)-28.6 min (50/45/5). The flow rate was 0.35 ml/min and the column temperature was 47°C. The injection volume was 5 μl.

Comprehensive LC-SRM-MS analysis was performed in the positive ion mode electrospray ionization with the transitions [M + H]^+^ →184 for PC and [M + H]^+^ →[M + H-141]^+^ for PE to detect all diradyl PC and PE species possessing two even number carbon chains each 14–24 carbons in length. Peak areas of all identified species within PC or PE were summed to obtain the total PC or PE signal. Peak areas of individual PC or PE species were normalized to this sum, and peak values were expressed as the percentage of the total. For each analysis, the seven most abundant peaks and all major species beyond this which comprised at least 5% of total PC or PE signals in any group were plotted. Although plasmalogen-type PC and PE are abundant in various tissues, they were not detected above the criteria we set to be plotted in our graphs, possibly due to inefficient detection under our assay conditions (Zemski Berry and Murphy, 2004).

### Semi-Quantitative qPCR

Total RNA was isolated from tissues using RNeasy and RNeasy fibrous tissue Kits (Qiagen, Valencia, CA, United States). cDNAs were synthesized using random primers and SuperScript III reverse transcriptase (Invitrogen, Carlsbad, CA, United States) or High-Capacity cDNA Reverse Transcription kit (Applied Biosystems, Waltham, MA, United States). Semi-quantitative qPCR analysis was performed using a StepOnePlus Real-Time PCR System and Fast SYBR Green Master Mix (Applied Biosystems, Waltham, MA, United States). mRNA expression was normalized to 18S rRNA and fold-changes were calculated using the 2^–ΔΔ^ *^CT^* method. Gene expression levels in EDL, GAS, and SOL were compared to EDL (values arbitrarily set to one) to determine statistical variations among the muscle types. The qPCR primer sequences are shown in Table 2.

**TABLE 2:** List of primers used for qPCR.

Gene	Forward primer	Reverse primer
*Agpat1*	AAACGAGGCGCCT TCCA	GGAGTAGAAGTCTTGATAGGAG GACATG
*Agpat2*	TGTGGGCCTCATCATGTACCT	AGGTCGGCCATCACAGACA
*Agpat3*	AAGCACCTATACCGCCGTATCA	GACCACCACTCCAGGAGCAT
*Agpat4*	AAGCAGCTGTTCCGCAAGA	CCACCACTCCAGAAGCATCA
*Agpat5*	AATGAGAAAGGTTCAGGAAAAT ACTCA	TGAATATGAAGTTTTGGGC ACTGT
*Tnnc1*	CTGTGAGCTGTCGCCAGAATG	CAGCATCCTCATCACCTTGCC
*Tnni1*	CCTAGCTCCACGAGGACTAAAC	CTGCTCCCAACACTCCTTGG
*Tnnt1*	GGACCCAGCCTTAGGTCTCT	CCCTTCTGGAATCTTCGGGG
*Tnni2*	TGCAAACAACTGCATGCGAA	TTGAACTTGCCCCTCAGGTC
*Tnnc2*	ATGGTGCGCCAGATGAAAGA	CCCAGAAGCCCGGAAAATCT
*Ppara*	CGTGGTGCATTTGGGCGTAT	CCATGTTGGATGGATGTGGC
*Ppard*	TCTCCCAGAATTCCTCCCCT	GAGCTTCATGCGGATTGTCC
*Pparg*	TGACAGGAGCCTGTGAGACC	GAATGGCATCTCTGTGTCAACC
*Pgc1a*	GGTGTAGCGACCAATCGGAA	TCTTCATCCACGGGGAGACT
*18S*	CTCAACACGGGAAACCTCAC	AGACAAATCGCTCCACCAAC

### Western Blots

Frozen muscle sections were homogenized in 2 × SDS-gel-loading buffer (4% SDS, 20% glycerol, and 0.125 M Tris pH to 6.8) by four cycles of bead-bashing, each for 120 s at 2,500 rpm, in a refrigerated Bead Smash12 (Wakenyaku). Cellular debris was pelleted at 12,000 × g for 15 min at room temperature; the supernatants were diluted 1:1 with water and protein concentrations were determined using a Pierce BCA Protein Assay kit (Thermo Fisher Scientific, Waltham, MA, United States). Then, 2-mercaptoethanol (5% final concentration) and bromophenol blue were added to the samples. After incubation at 95°C for 5 min, proteins (15 μg) were resolved by 4–20% SDS-PAGE and then transferred to Immobilon-P transfer membranes (Millipore, Burlington, MA, United States) using a semidry transfer cell. Membranes were blocked in 5% ECL Prime Blocking Agent in TBS containing 0.1% Tween 20 (TBS-T) and probed with anti-TnnI1 (Proteintech #16102-1-AP, Rosemont, IL, United States) or anti TnnI2 (Proteintech #22130-1-AP, Rosemont, IL, United States) rabbit polyclonal antibodies. After incubation at room temperature for 60 min, the blots were washed three times for 5 min each with TBS-T and then incubated with horseradish peroxidase-conjugated anti-rabbit IgG (Cytiva #NA9340, Marlborough, MA, United States) for 30–60 min. Detection was performed with ECL Prime Western Blotting Detection Reagent (GE Healthcare, Chicago, IL, United States) and imaged using a Bio-Rad ChemiDoc MP Imaging System. Following detection, the blots were incubated for 15 min in Restore PLUS Western Blot Stripping Buffer (Thermo Fisher Scientific, Waltham, MA, United States) and re-blocked overnight then probed with anti-alpha-Tubulin antibody (Sigma #T6199, St. Louis, MO, United States) followed by horseradish peroxidase-conjugated anti-mouse IgG (Cytiva #NA9310, Marlborough, MA, United States).

### Animal Diets

For studies comparing effects of feeding CE-2 standard chow (CLEA Japan, Inc., Tokyo, Japan) to custom research diets (Research Diets, Inc., New Brunswick, NJ, United States), diets were fed from the first day after birth, starting with to their nursing mothers (Oosting et al., 2015). The custom diet formulations are based on the common AIN-93G rodent growth diet, but with the fat sources substituted so as to modify the fatty chain compositions to be relatively rich in FA 18:1/oleic acid chains or FA 18:2/linoleic acid chains. Unlike the CE-2 standard chow which contains appreciable levels of FA 20:5/eicosapentaenoic acid (EPA) and FA 22:6/docosahexaenoic acid (DHA) fish oils, both custom diets lack appreciable levels of EPA and DHA, and they differ from CE-2 standard chow in nutritional energy contents as well. Estimates of the fatty chain compositions of CE-2 standard chow and both custom diets are shown in Table 3 and the nutritional estimates are shown in Table 4, and nutritional estimates of the AIN-93G diet are also included for reference. Fatty chain composition estimates of custom research diets were reported by the manufacturer, and the fatty chain compositions of CE-2 standard chow were determined by GC-FID. Traditional Atwater formula was used to calculate estimation of metabolizable energy (ME): ME(kcal/kg) = [4 × CP(%) + 4 × NFE(%) + 9 × crude fat(%)] × 10 (Bielohuby et al., 2010; Asaro et al., 2017), based on the nutritional components reported by the manufacturers.

**TABLE 3:** Estimation of fatty acid compositions of diets (% by weight of total fatty acids).

	CE-2 chow	Oleic custom diet	Linoleic custom diet
FA 16:0	18.2	12.8	13.2
FA 16:1	1.0	0.1	0.1
FA 18:0	2.5	3.8	3.2
FA 18:1	22.3	55.0	19.7
FA 18:2	46.0	24.2	59.6
FA 18:3	3.4	3.0	3.1
FA 20:5	2.4	0.0	0.0
FA 22:6	2.2	0.0	0.0
Saturates	21.6	17.6	17.4
Monounsaturates	23.8	55.1	19.9
Polyunsaturates	54.6	27.2	62.7
n-3	8.5	3.0	3.1
n-6	46.2	24.2	59.6

*Fatty acids comprising ≥ 0.5% in any diet are shown. Custom diet values reported by manufacturer. CE-2 standard chow values measured by GC-FID.*

**TABLE 4:** Nutrition of diets.

	CE-2 chow	Custom diets	AIN-93G
kcal% protein	30.1	20.1	20.3
kcal% fats	12.2	15.8	15.8
kcal% carbs	57.7	64.2	63.9
kcal/100 g	339	399	399

*Based on nutritional components reported by manufacturers. Energy contents were estimated by Atwater equations. AIN-93G is shown for reference.*

### Statistical Analysis

Reverse transcription (RT)-PCR expression values were log-transformed before statistical analyses (Derveaux et al., 2010). All *t*-tests (unpaired, 2-tailed), Dunnett’s multiple comparison tests, and two-way ANOVA with Sidak’s post-tests were performed as indicated in figure legends. For multiple unpaired *t*-tests (more than two), the false discovery rate (FDR) was controlled by the Benjamini, Krieger, and Yekutieli method (FDR < 5%). All statistical analyses were performed using GraphPad Prism 9 software (GraphPad Software, La Jolla, CA, United States).

## Results

### Phosphatidylcholine and Phosphatidylethanolamine Profiles of Healthy Skeletal Muscles of C57BL/6 Mice

To determine the variables, other than loss of dystrophin, which may cause PC 34:1/PC 34:2 ratios in muscles to differ between studies, we first examined how phospholipid profiles vary in different muscles of healthy adults C57BL/6 mice. Initial studies indicated that PC and PE compositions were dynamically regulated until age 6-weeks but highly stable in the adult tissues from ages 12- until at least 24-weeks, thus we analyzed muscle tissues from 15-week-old C57BL/6 mice. We consider this timepoint and others between 12- and 18-weeks of age nearly equivalent for adult mouse muscle tissues in terms of their phospholipid profiles. We measured PC and PE compositions in four muscles that vary in their metabolic capacities—tibialis anterior (TA), EDL, GAS, and SOL, with SOL being the most oxidative. The peak areas of each PC or PE species were normalized against the sum of all peak areas within that class to determine the relative abundances (expressed as a percent of total PC or PE), and all major species comprising at least 5% of total PC or PE signals were plotted. Of note, plasmalogen (ether-linked) PC and PE were not detected at levels above the 5% threshold, but this may be due to their underestimation under our assay conditions (Zemski Berry and Murphy, 2004).

**FIGURE 1:**
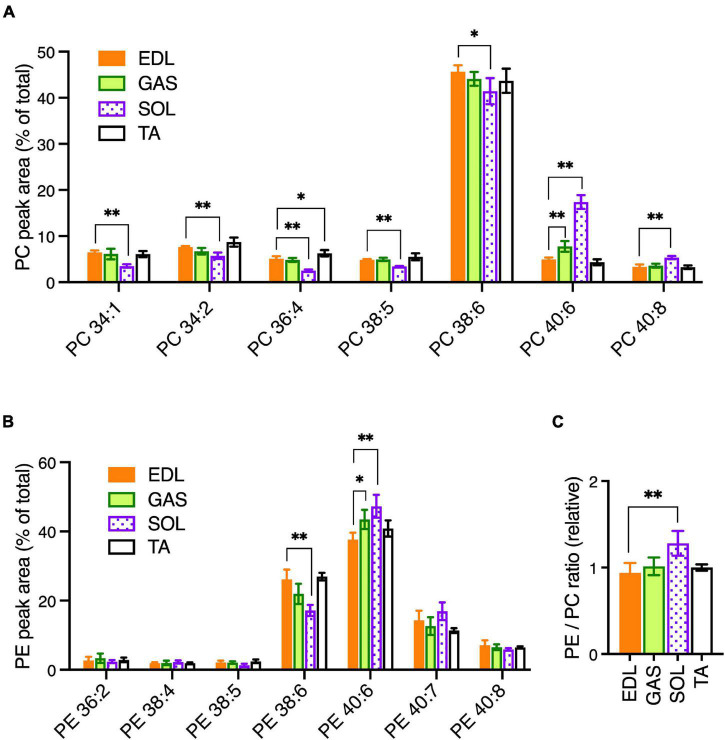
PC and PE profiles in healthy EDL, GAS, SOL, and TA skeletal muscles of adult (15-week-old) C57BL/6 mice raised on CE-2 standard chow. PC **(A)** and PE **(B)** were measured by LC-MS/MS. EDL was selected as a standard muscle to determine the statistical variations. **(C)** Relative ratios of total detected amounts of PE to PC (ratio in EDL = 1). PC and PE peak values are expressed as the percentage of total PC or PE signals and means ± *SD* are plotted. The statistical significance of variations vs. EDL is based on Dunnett’s multiple comparison tests. *p < 0.05, ^**^p < 0.01; *n* = 4 mice/group.

To determine the statistical variations of PC and PE compositions among the muscle types, EDL muscle was selected as a “standard” muscle to compare the variations that occurred in GAS, SOL, and TA. GAS and TA had more similar PC and PE profiles to EDL, and SOL had more pronounced variations. In PC (Figure 1A), relatively modest variations with EDL were detected in GAS (increased PC 40:6) and TA (increased PC 36:4). SOL showed substantially more variation with EDL, with SOL having decreased PC 34:1, PC 34:2, PC 36:4, PC 38:5, and PC 38:6; and increased PC 40:6 and PC 40:8. The most pronounced variation of PC in SOL was the markedly high levels of PC 40:6, which includes FA 22:6/DHA-containing PC 18:0_22:6 as an abundant isomer in muscle cells (Valentine et al., 2018).

In PE (Figure 1B), GAS had high levels of PE 40:6 compared to EDL. This same variation was more pronounced in SOL, which also varied from EDL in having low levels of PE 38:5. The four most abundant PE peaks—PE 38:6, PE 40:6, PE 40:7, and PE 40:8—may all contain DHA in their fatty chain sets, suggesting PE as a class may have been rich in DHA. To determine if PE might, as a class, be relatively enriched in any of the muscles, we calculated the ratios of total PE to total PC (sum of all PE peak areas/sum of all PC peak areas). While GAS and TA had total PE/total PC ratios that were similar to the ratio in EDL, the ratio in SOL was higher (Figure 1C). Together these data indicate that notable variations occurred in SOL that includes elevated levels of the DHA-containing peak PC 40:6 and elevated levels of PE, which as a class may have been rich in DHA-containing peaks. Notably, these mice were raised on a standard rodent diet (CE-2 standard chow) that has appreciable levels of DHA and EPA fish oils (Table 3). This is also likely to influence the prominence of DHA-containing peaks in PC and PE profiles because dietary DHA intake influences the amounts of DHA-containing phospholipids in tissues (Calder, 2016). It is also important to note that plasmalogen-type PC and PE species may have been inefficiently detected in our data-set but may also contain significant amounts of DHA chains.

### Metabolic Gene Expression in Skeletal Muscles of C57BL/6 Mice

**FIGURE 2:**
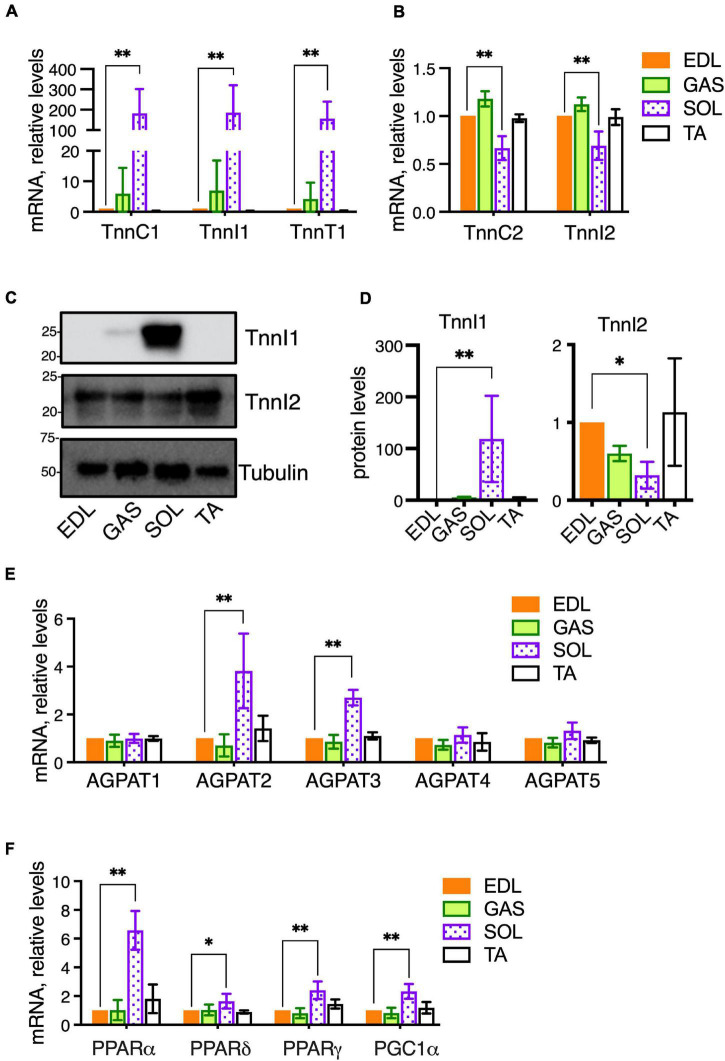
Metabolic gene expressions in healthy skeletal muscles of adult (14–16-week-old) C57BL/6 mice raised on CE-2 standard chow. mRNA levels were measured by qPCR and protein levels (for TnnI1 and TnnI2) were detected by Western blotting. EDL was selected as a “standard” muscle to determine the statistical variations, and the plotted values reflect relative abundances compared to the levels in EDL (arbitrarily set to “1”). Compared to EDL, SOL had markedly high levels of slow-type troponins TnnC1, TnnI1, and TnnT1 **(A)** and moderately decreased fast-type troponins TnnC2 and TnnI2 **(B)**. Similar patterns in SOL were observed for the protein levels of TnnI1 (high levels) and TnnI2 (moderately decreased) **(C,D)**. mRNA levels of AGPATs **(E)** and PPARs/PGC1-alpha transcriptional regulators **(F)** were also determined. mRNA levels were normalized to 18S RNA and protein levels were normalized to Tubulin. The means ± *SD* are plotted. Statistical significance is based on Dunnett’s multiple comparison tests. *p < 0.05, ^**^p < 0.01; *n* = 4 mice/group.

Different skeletal muscle groups have different embryonic origins and acquire different physiological characteristics during development depending on their physiological roles to generate rapid bursts of motion or more sustained but less rapid motion. Accordingly, the compositions of fiber types within muscles vary in terms of fast-twitch vs. slow-twitch fibers and glycolytic vs. oxidative fibers. In mice, SOL contains a majority (>75%) of oxidative fibers, both slow-twitch (Type I) and fast-twitch (Type IIA), while TA, EDL, and GAS, contain primarily fast-twitch, glycolytic fibers (Type IIB and IIX) (Augusto et al., 2004; Talbot and Maves, 2016; Terry et al., 2018). It is possible the distinctive PC and PE features we detected in SOL may be related to the metabolic characteristics of this muscle, because high phospholipid-DHA levels and high PE levels may be associated with high oxidative metabolic capacity in muscle (Hishikawa et al., 2017; Heden et al., 2019). Thus, we measured the mRNA expression of metabolic-related genes in the muscles of the same 15-week-old C57BL/6 mice that had been used for the comparative analyses of PC and PE compositions between EDL, GAS, SOL, and TA. In comparing the expression of genes among the muscle types that are involved in the metabolic regulation of membrane compositions, we first measured the gene expressions of several troponins as markers of fast- and slow-twitch fibers (Figures 2A,B). EDL was again used as a “standard” muscle to determine the statistical variations. SOL was markedly enriched in mRNA for the slow-type troponins TnnC1, TnnI1, and TnnT1 (Figure 2A). SOL also showed moderately reduced mRNA levels of the fast-type troponins TnnC2 and TnnI2 (Figure 2B). In a similar cohort of 14- to 16-week-old mice, proteins levels were analyzed. Similar to the mRNA data, SOL also had high protein levels of slow-type TnnI1, while fast-type TnnI2 protein was moderately decreased in SOL compared to EDL (Figures 2C,D).

Our lipidomic data had shown that SOL had high levels of DHA-containing PC 40:6 and was also possibly rich in DHA-containing PE (Figures 1A–C). Levels of PC 40:6 and several DHA-containing PE species were previously shown to be regulated in muscle cells by 1-acyl-*sn*-glycerol-3-phosphate O-acyltransferase 3 (AGPAT3), also called LPAAT3, an enzyme that selectively incorporates DHA into phospholipids (Valentine et al., 2018). To assess whether SOL may have enhanced levels of AGPAT3 or other enzymes that may regulate PC and PE compositions, we measured the expression of four AGPAT enzymes (AGPAT1-4) and another candidate enzyme (AGPAT5) which may regulate fatty chain compositions during *de novo* synthesis of phospholipids (Figure 2E; West et al., 1997; Lu et al., 2005; Shindou and Shimizu, 2009; Yuki et al., 2009; Eto et al., 2014). Consistent with our hypothesis, AGPAT3 expression was highest in SOL, which also showed higher levels of AGPAT2, compared to the other muscles (Figure 2E). While AGPAT3 is highly selective to utilize DHA as substrate and generate DHA-containing phospholipids (Koeberle et al., 2012; Iizuka-Hishikawa et al., 2017; Shindou et al., 2017), it is unknown how enhanced levels of AGPAT2 might impact PC and PE compositions in tissues. Major physiological roles in lipid metabolism are known for AGPAT2 (Agarwal et al., 2002; Cortes et al., 2009), and its high levels in SOL might be related to the high metabolic capacity of this muscle.

We also examined mRNA levels of peroxisome proliferator-activated receptors (PPARs) and PPAR Gamma Coactivator-1α (PGC-1α), which are ligand-activated transcriptional regulators of cellular metabolism (Figure 2F). PPARα, PPARδ, and PGC1α are major transcriptional regulators of genes involved in oxidative metabolism, and PPARγ is a transcriptional regulator of glucose metabolism. While these genes were expressed in GAS and TA at similar levels as EDL, the levels in SOL were higher. PPARδ and PGC1a cooperatively promote oxidative metabolism and increase endurance in skeletal muscle (Fan and Evans, 2017; Phua et al., 2018). We previously reported that PPARδ and PGC1α agonists lead to enhanced levels of DHA-containing PC and PE in skeletal muscle cells through upregulation of AGPAT3 (Valentine et al., 2018), and it is possible that a similar PPAR pathway contributes to high levels of AGPAT3 and DHA-containing phospholipid in SOL. Overall, EDL and GAS muscles showed similar patterns of gene expression to EDL, while the patterns in SOL varied markedly (Figures 2A–F). Together with the lipidomic analyses (Figures 1A–C), these results support that the markedly different metabolic character of SOL compared to EDL, GAS, and TA is also reflected in PPAR-signaling pathways that impact fatty acid compositions of muscle cell membranes.

### Phosphatidylcholine and Phosphatidylethanolamine Profiles in Skeletal Muscles of Wild-Type and *Mdx* Mice

In *mdx* mice, myofiber plasma membrane may be similarly destabilized as in DMD, and it has been suggested that similar PC alterations also occur. In order to determine the specific alterations of *mdx* mouse muscles, we measured PC and PE compositions of WT and *mdx* muscles by LC-MS/MS. All tissues used for comparative analyses of WT vs. *mdx* muscles were from B10 strain male mice, and all mice were raised on CE-2 standard chow diets, except in special cases for custom diet studies.

**FIGURE 3:**
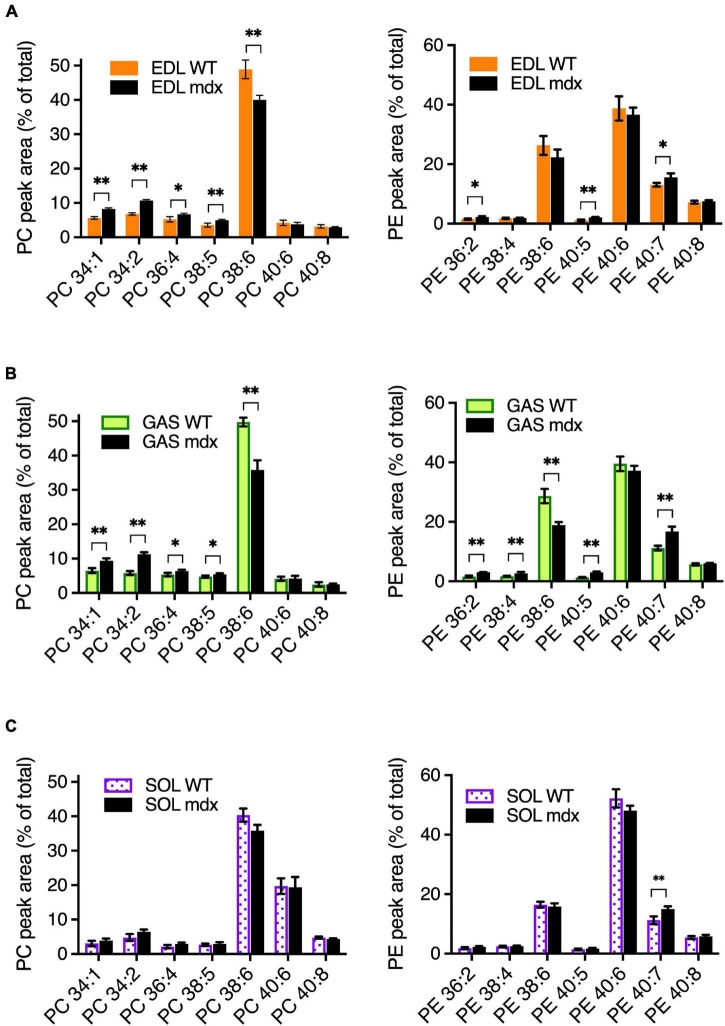
*Mdx*-related PC and PE alterations in EDL, GAS, and SOL of adult (18-week-old) B10-WT and -*mdx* mice raised on CE-2 standard chow. EDL **(A)** and GAS **(B)** showed similar patterns of *mdx*-associated PC alterations. PC alterations were diminished in SOL **(C)**. The most prominent *mdx*-associated PC alterations in EDL and GAS were high PC 34:1, high PC 34:2, and reduced PC 38:6 levels. *Mdx*-associated alterations in PE are also shown. PC and PE were measured by LC-MS/MS. Peak values are expressed as the percentage of total PC or PE signals and means ± *SD* are plotted. Statistical significance of corresponding WT and *mdx* tissues is based on FDR-controlled multiple *t*-tests (FDR < 5%). *p < 0.05, ^**^p < 0.01; *n* = 3–5 mice/group.

To assess the *mdx*-specific alterations in adult mouse muscles, PC and PE compositions were measured in EDL, GAS, and SOL of 18-week-old adult WT and *mdx* mice (Figures 3A–C). The patterns of muscle-type variations in PC and PE between EDL, GAS, and SOL in these tissues (Supplementary Figures 1A–C) were similar to those observed in the 15-week-old WT C57BL/6 tissues (Figures 1A–C), consistent with our observations that PC and PE compositions are relatively stable in adult mouse muscle tissues between from ages 12-weeks to at least 24-weeks, and also indicating that mouse strain has little influence in influencing the PC and PE profiles between B10-WT and C57BL/6 mice. As for the *mdx*-specific alterations that were detected in comparative analyses of the adult B10-WT and B10-*mdx* mice, EDL and GAS showed similar patterns in PC (Figures 3A,B; left graphs), with the *mdx*-associated PC alterations including high PC 34:1, high PC 34:2, high PC 36:4 high PC 38:5, and reduced PC 38:6. In SOL; however, these *mdx*-associated alterations were diminished below significance (Figure 3C, left graph). PE profiles were also measured (Figures 3A–C, right graphs), and several *mdx*-specific alterations were detected. PE 40:7 was moderately higher in *mdx* in all three muscle types, and other alterations varied among the muscles. Overall, the PC and PE patterns were more similar in EDL and GAS but varied somewhat in SOL. Transcriptional profiling studies (Terry et al., 2018) indicated that EDL and GAS had high transcriptome similarity with TA and several other skeletal muscles (quadriceps, plantaris, and masseter), while SOL had similarity with a different cluster that included the diaphragm. Our data suggest that muscles like TA, EDL, and GAS may also cluster in terms of phospholipid profiles and *mdx*-specific alterations and indicate that muscle type sampled is one factor that affects the detection of PC alterations in dystrophic muscle.

### Time Course of Alterations of Phosphatidylcholine and Phosphatidylethanolamine in *Mdx* Muscles

The muscle pathology in *mdx* mice proceeds in stages. Muscles of neonate *mdx* mice lack signs of overt muscle pathology before the initial wave of degeneration occurs at age ∼ 3-weeks (Supplementary Figures 2A,B; DiMario et al., 1991; Pastoret and Sebille, 1995). In *mdx* mice of ∼ 3-weeks of age, clusters of muscle fibers undergo initial rounds of necrosis and regeneration, evident in muscle tissue sections as regions populated by degenerated and small reforming fibers. Both WT and *mdx* mouse muscles grow rapidly from ages 3- to 6-weeks and are nearly fully grown by age 12-weeks. Abundant centrally located nuclei within myofibers indicate the active regeneration in adult (i.e., 6- and 12-week-old) *mdx* muscles (Supplementary Figure 2C).

**FIGURE 4:**
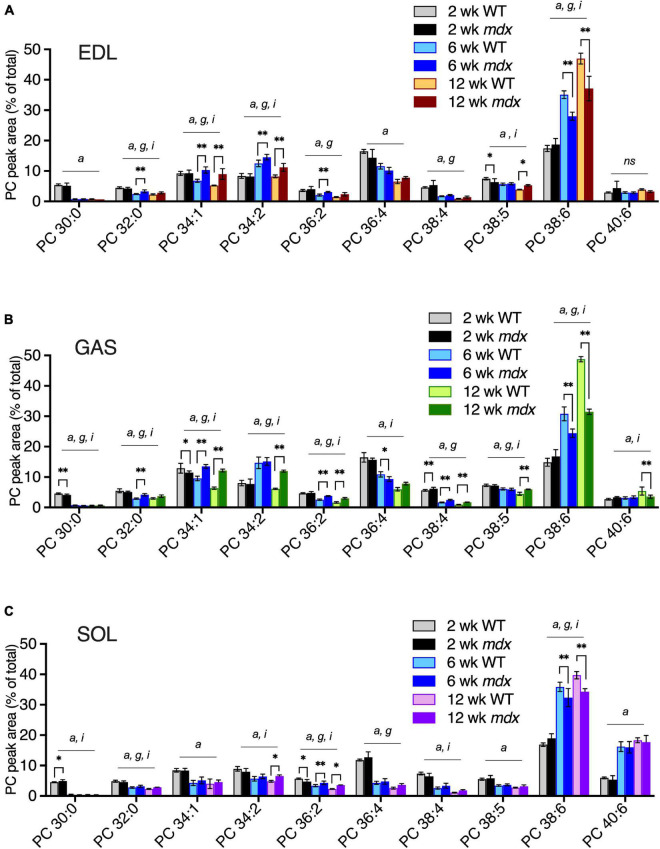
PC alterations in EDL, GAS, and SOL of B10-WT and -*mdx* mice of various ages (2-, 6-, and 12-week-old) were raised on CE-2 standard chow. EDL **(A)** and GAS **(B)** showed similar patterns of *mdx*-associated PC alterations for increased PC 34:1 and decreased PC 38:6 at ages 6- and 12-weeks, and increased PC 34:2 at age 12-weeks, as well as at age 6-weeks in EDL. *Mdx*-associated PC alterations in SOL **(C)** were diminished or absent in several cases. PC peak values are expressed as the percentage of total signals and means ± *SD* are plotted. Significant variation (*p* < 0.05) was determined by two-way ANOVA and factor effects for each peak are indicated for age (*a*), *mdx* genotype (*g*), and interactive effects (*i*). Significant pair-wise differences between same-aged WT and *mdx* muscles were determined by Sidak’s post-tests; *p < 0.05, ^**^p < 0.01. *n* = 3–6 mice/group. *ns*; non-significant.

We examined how the *mdx*-alterations in EDL, GAS, and SOL correlated with the time course of *mdx* muscle pathology. PC compositions in 2-, 6-, and 12-week-old muscles were examined in order to determine whether PC alterations detected in mature adult muscles (i.e., ages 12-weeks) also occurred before muscle disease onset (age 2-weeks) or in young adult muscle undergoing maximum growth (age 6-weeks) (Figures 4A–C). Similar to the *mdx*-alterations detected in 18-week-old EDL and GAS muscles (Figure 3A), in 12-week-old EDL and GAS (Figures 4A,B) *mdx*-associated high PC 34:1 and high PC 34:2 were observed, consistent with our observations that adult PC and PE profiles are fully developed and stable in WT and *mdx* mice by age 12-weeks. At age 6-weeks, high *mdx*-levels of PC 34:1 were also evident in EDL and GAS, however PC 34:2 was also transiently increased in WT muscles at age 6-weeks and additional *mdx*-associated elevations of PC 34:2 were modest (in EDL) or not detected (in GAS) at this time point. The other major *mdx*-associated alterations of PC, reduced PC 38:6, was evident in all muscles at age 6- and 12-weeks, but the alteration was diminished in SOL compared to EDL and GAS (Figures 4A–C). *Mdx*-alterations detected in adult muscles at ages 6- or 12-weeks were largely not present at age 2-weeks before the onset of muscle pathology (high PC 38:4 in GAS was an exception), and other minor *mdx*-associated PC alterations detected at age 2-weeks (i.e., in PC 30:0 in GAS or SOL) might reflect roles of dystrophin during muscle development (Dumont et al., 2015). PE compositions were also examined, and the adult *mdx*-associated alterations detected by age 6- or 12-weeks were not detected at age 2-weeks (Supplementary Figure 3).

**FIGURE 5:**
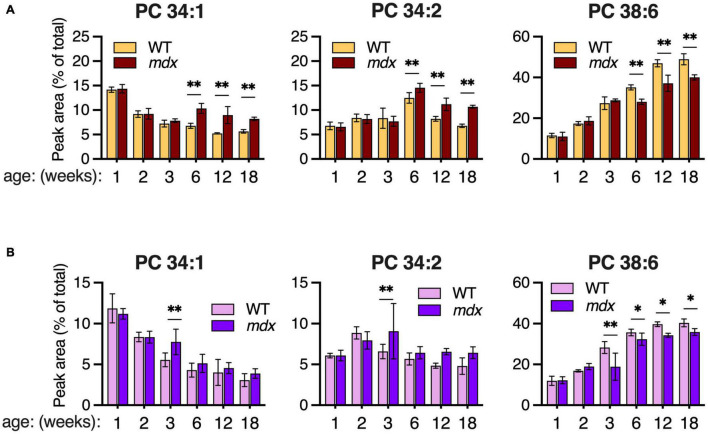
Time course of major PC alterations in EDL and SOL of 1–18-week-old B10-WT and -*mdx* mice raised on CE-2 standard chow. PC compositions were measured by LC-MS/MS and means ± *SD* are plotted. In EDL **(A)**, high *mdx*-levels of PC 34:1 and PC 34:2 and reduced *mdx*-levels of PC 38:6 were detected at ages 6-, 12-, and 18-weeks. In SOL **(B)**, these alterations tended to be detected earlier at age 3-weeks and were diminished at later time points. Significant differences between corresponding WT and *mdx* muscles were determined by FDR-controlled multiple *t*-tests (FDR < 5%). *p < 0.05, ^**^p < 0.01; *n* = 3–8 mice/group; includes identical data shown in Figure 3 (18-week-old) and Figure 4 (2-, 6-, and 12-week-old).

Although PC 34:1 and PC 34:2 were similarly enhanced in 12-week-old *mdx* EDL and GAS muscles (Figures 4A,B), the pattern of change in WT muscles between ages 2- to 6-weeks was opposite with PC 34:1 decreasing and PC 34:2 increasing. These two abundant PC species are of great interest because they may harbor a large proportion of the FA 18:1 and FA 18:2 chains that are reportedly altered in the PC of DMD patients’ muscles. Reduced PC 38:6 was also one of the most pronounced PC alterations we detected in *mdx*-muscles, and the extended time course of the dynamic change in all three species was examined in EDL and SOL. High *mdx*-levels of PC 34:1 and PC 34:2, and reduced *mdx*-levels of PC 38:6 were detected in EDL at ages 6-, 12-, and 18-weeks (Figure 5A). These same *mdx*-alterations were generally diminished in SOL (Figure 5B); however, surprisingly the time course of these alterations in SOL was also different, with all three alterations maximally detected earlier in SOL, at age 3-weeks, and then diminished or absent at later time points.

### Phosphatidylcholine and Phosphatidylethanolamine Profiles in Extensor Digitorum Longus Muscles of Wild-Type and *Mdx* Mice Raised on Different Diets

While elevated levels of PC 34:1 in *mdx* muscles were consistently reported by several studies, altered levels of PC 34:2 are variably reported to occur. Dietary fats strongly influence fatty chain compositions of phospholipids in tissues (Levental et al., 2020); therefore, we examined how dietary factors may affect *mdx*-specific PC and PE alterations. In addition to CE-2 standard chow (CLEA, Japan, Inc.), we also raised mice on either of two custom diets that were formulated based on the commonly used AIN-93G rodent diet but with the sources of fats adjusted so as to be relatively high in FA 18:1 or FA 18:2. The fatty chain compositions and nutritional estimates of all three diets are shown in Tables 3, 4. CE-2 standard chow differs from both custom diets in nutritional contents and also in being relatively rich in EPA and DHA compared to the custom diets; however, all diets contain at least 3% of fatty chains as FA 18:3 (n-3) and provide adequate amounts of essential fatty acids including omega-3s.

**FIGURE 6:**
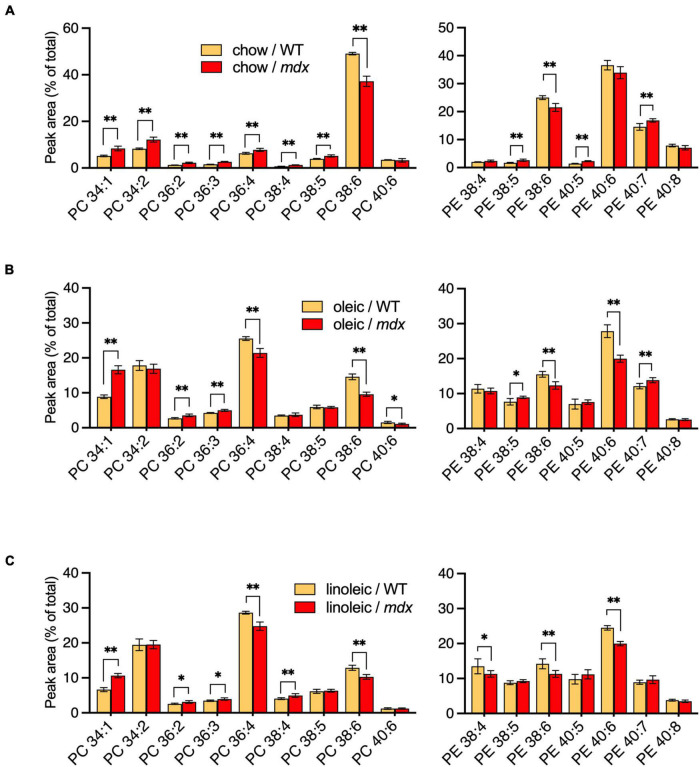
PC and PE profiles of EDL from 12-week-old B10-WT and -*mdx* mice fed different diets from birth. Mice were raised on CE-2 standard chow (chow) **(A)**, or custom diets rich in FA 18:1 (oleic) **(B)** or FA 18:2 (linoleic) **(C)**. PC compositions were measured by LC-MS. PC 34:1 showed a similar increase in *mdx* tissue regardless of diet, but increased PC 34:2 was variably detected in *mdx* tissues, depending on the diets. PC and PE peak values are expressed as the percentage of total signals and means ± *SD* are plotted. Significance is based on FDR-controlled multiple *t*-tests (FDR < 5%). *p < 0.05, ^**^p < 0.01; *n* = 4–8 mice/group.

PC and PE compositions were measured in EDL muscles of adult WT and *mdx* mice raised on CE-2 standard chow (chow) (Figure 6A), the high-FA 18:1 custom diet (oleic) (Figure 6B), or the high-FA 18:2 custom diet (linoleic) (Figure 6C). The diets were provided to the mice from birth, starting with being fed to their nursing mothers, and tissues were collected at 12-weeks of age, as at this age the PC and PE profiles of the muscles were stable and fully matured, both in WT and *mdx* tissues. Diets profoundly affected the PC and PE compositions regardless of *mdx* status, with the most evident diet-dependent differences being observed between CE-2 standard chow and the custom diets. Some of these differences may reflect the relative lack of DHA in the custom diets, such as the large reduction in the presumed DHA-containing PC 38:6 peak and a corresponding increase in the non-DHA-containing PC 36:4 peak, which was observed in muscles of custom diet-raised mice (Figures 6B,C) compared to chow-raised mice (Figure 6A).

Several of the *mdx*-specific alterations in the major PC peaks were influenced by the diets to various extents. High-*mdx* levels of PC 34:1 were consistently observed in adult EDL regardless of the diet. In contrast, alterations of PC 34:2 were strictly diet-dependent, and high-*mdx* levels were only detected if mice were raised on CE-2 standard chow (Figure 6A) but not if mice were raised on either custom diet (Figures 6B,C). The low-*mdx* levels of PC 38:6 were also consistently observed in *mdx* muscles regardless of diet, although the overall abundance of this peak was much lower in custom diet-raised mice compared to CE-2 standard chow, suggesting the PC species in this peak may be replaced or compensated for by other species, possibly in the PC 36:4 peak, in a diet-dependent manner. Both the PC 36:4 and PC 38:6 peaks are likely to contain mixtures of isomers including those that contain FA 18:2 in their chain sets, which will be important to investigate in future studies.

We also examined how the PC 34:1, PC 34:2, and PC 38:6 levels varied during early growth and development (ages 1–3-weeks) compared to those seen in adult EDL tissues of mice raised on the custom diets (Supplementary Figures 4A,B). During the first 3 weeks following birth, the pattern of changes in all three peaks was similar to those we had observed in EDL of chow-raised mice (Figure 5A), with no *mdx*-specific alterations being apparent. PC 34:1 was high in neonates and decreased until age 3-weeks, while PC 34:2 and PC 38:6 tended to increase until age 3-weeks. The pattern of alterations detected at age 12-weeks of PC 34:1 (high in *mdx*) and PC 38:6 (low in *mdx*) were also qualitatively similar in all three diets (Figures 6A–C); however, lack of any *mdx*-specific elevation of PC 34:2 at age 12-weeks in custom-diet raised mice indicates that the high PC 34:2 levels observed in chow-raised *mdx* muscles is not only *mdx*- but also diet-dependent, possibly reflecting increased nutritional demands in the regenerating fibers and distinguishing it from the high levels of PC 34:1 that are diet-independent features of regenerating *mdx* muscles.

### Metabolic Gene Alteration in Extensor Digitorum Longus Muscles of Adult *Mdx* Mice

We have shown that the mRNA expression profiles of various genes involved in metabolism in skeletal muscles of wildtype mice are markedly different in SOL when compared to the other muscles examined (EDL, GAS, and TA) (Figures 2A–F), which correlated with altered phosphatidylcholine and phosphatidylethanolamine compositions (Figures 1A,B). It was therefore critical to determine whether mRNA expression profiles of the metabolic genes are also altered in muscles of *mdx* mice and correlate with the altered lipid profile of *mdx* muscle tissue. We profiled the expression levels of fast type troponins and slow type troponins, AGPATs, and PPAR/PGC1-alpha transcriptional regulators in the EDL tissues of adult WT and *mdx* mice (chow-raised) in order to document the differences, if any, that exist in the muscles of *mdx* mice and correlate with the altered lipid profiles.

**FIGURE 7:**
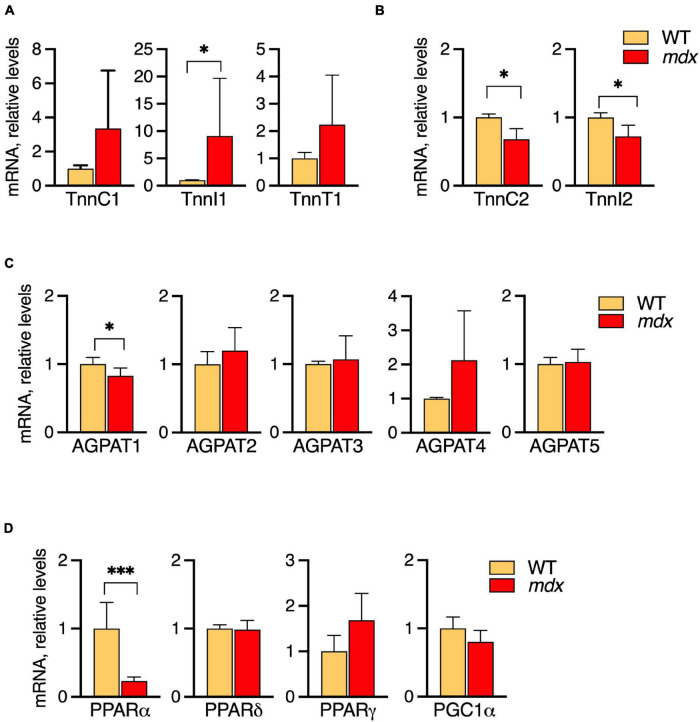
Profiles of mRNA levels of metabolic genes in EDL of 12-week-old B10-WT and -*mdx* mice raised on CE-2 standard chow. mRNA levels were measured by qPCR and the abundances in *mdx* tissues relative to those in WT are plotted. High levels of slow-type troponin TnnI1 were observed in the *mdx* tissues **(A)** while fast-type troponins TnnC2 and TnnI2 were decreased in *mdx* tissues **(B)**. Among several AGPATs, AGPAT1 was slightly decreased in the *mdx* tissue **(C)**. Among several PPAR signaling molecules, PPARα was markedly down regulated in the mdx tissue **(D)**. The means ± *SD* are plotted. Significance is based on *t*-tests. *p < 0.05, ^***^p < 0.001; *n* = 4–5 mice/group.

Extensor digitorum longus (EDL) muscles of 12-week-old WT and *mdx* mice were selected for the comparative analyses because the characteristic *mdx*-specific PC alterations are fully manifested by this age, and mRNA levels of the metabolic genes were measured by qPCR (Figures 7A–D).

Among the troponin genes analyzed, elevated levels of slow-type troponin TnnI1 were detected in the *mdx* tissues (Figure 7A) while fast-type troponins TnnC2 and TnnI2 were decreased in *mdx* tissues (Figure 7B). Among the AGPAT1-5, AGPAT1, a molecule involved in phospholipid and triglyceride synthesis, was slightly decreased in the *mdx* tissue (Figure 7C). Among several PPAR/PGC1α transcriptional regulators, PPARα was markedly down regulated in the *mdx* tissue (Figure 7D). Besides these alterations, other tendencies were noted, i.e., levels of the slow troponins TnnC1 and TnnT1 also tended to increase in *mdx* tissues, although not reaching statistical significance. Overall, these results indicated that EDL muscles of *mdx* mice do have altered metabolic gene expressions that accompany their altered lipid alterations. Some alterations such as increased slow-type and decreased fast-type troponin mRNA levels qualitatively resemble patterns seen in SOL, while other patterns differ such as in AGPATs and PPAR/PGC1α transcriptional regulators.

## Discussion

In DMD, myofiber membrane fragility caused by lack of dystrophin is a direct cause of the skeletal muscle pathology, and lipid alterations in DMD and the *mdx* mouse model have been long noted and are of great interest because of their possible impact on the disease course (for review see Saini-Chohan et al., 2012). Increase of FA 18:1 and decrease of FA 18:2 chains in PC of DMD patients muscles were noted in early studies (Takagi et al., 1968; Kunze et al., 1975; Pearce et al., 1981). In later studies, LC-MS/MS-based technologies were combined with coarse imaging capabilities to localize the PC alterations in tissues (Tahallah et al., 2008). PC 34:1, containing 16:0_18:1 chain sets, and PC 34:2, containing 16:0_18:2 chain sets, were detected as major PC species in muscle. Elevated PC 34:1/PC 34:2 ratios were detected in DMD muscle, with higher PC 34:1/PC 34:2 ratios detected in more affected areas both in regions of regenerating fibers as well as intercellular spaces (Tahallah et al., 2008). A more recent MS-imaging study also reported enhanced PC 34:1 as a prominent feature of DMD muscle (Dabaj et al., 2021), however, the relationship and impact of the enhanced PC 34:1 to the membrane fragility and progressive muscle wasting remains an open and pressing question.

Fatty acid (FA) 16:0, FA 18.1, and FA 18:2 were reported to constitute the majority of fatty chains in PC of normal human muscle throughout life, with FA 18:1 levels high in gestation and decreasing through development until adulthood, while FA 18:2 levels follow a reciprocal pattern of low in gestation and becoming high by adulthood (Bruce and Svennerholm, 1971). This observation suggests that PC 34:1 and PC 34:2 may be major PC species in human skeletal muscle throughout life, with PC 34:1 high in gestational and newborn muscle and decreasing through development as it is replaced by PC 34:2. In DMD muscle, replacement of FA 18:2 chains with FA 18:1 chain at high levels as reported in the early studies (Takagi et al., 1968; Kunze et al., 1975; Pearce et al., 1981) suggests that affected DMD muscle may reaquire a high PC 34:1/low PC 34:2 aspect that is also characteristic of the fetal and early development of muscle. As PC is the most abundant phospholipid (Vance, 2015) it is possible that the relative amounts of FA 18:1 and FA 18:2 chains in PC must be appropriately regulated in order to maintain appropriate biophysical properties of lipid bilayers critical for various aspects of muscle development and function. It has been shown that artificial lipid bilayers of giant PC liposomes (∼20-micron diameter) containing only FA 18:1 chains were less flexible but withstood twice as much tension force before rupture as PC liposomes containing only FA 18:2 chains (Olbrich et al., 2000; Rawicz et al., 2000). Thus it is conceivable that control of the fatty chains in membrane phospholipids might have a similar therapeutic potential as other membrane-stabilizing strategies being pursued, most notably for synthetic block copolymers such as Poloxamer 188, to treat DMD and other conditions where membrane integrity is compromised (Houang et al., 2018). Whether and to what extent FA 18:1 and FA 18:2 abundances may influence biophysical properties of biological membranes such as the sarcolemma of dystrophic myofibers is currently unknown and will require rigorous experimental model systems to be addressed.

Enhanced PC 34:1 also occurs in the skeletal muscle of *mdx* mice, indicating they may be a useful model to elucidate the biological meaning of any therapeutic potential of the PC alterations in DMD patients’ muscles. Touboul et al. (2004) used MALDI-TOF MS with course imaging capabilities to measure the PC 34:1/PC 34:2 ratios in structured (i.e., relatively healthy) vs. destructured (i.e., severely affected) areas of *mdx* mouse muscles. In this ground-breaking study, the PC 34:1/PC 34:2 ratios were higher in destructured areas of *mdx* muscle than in the relatively structured areas or healthy WT muscle. In an illuminating follow-up study (Benabdellah et al., 2009), the more destructured area of *mdx* muscles again had a higher PC 34:1/PC 34:2 ratio than in structured *mdx* muscles or WT, and a cohort of *mdx* mice was treated with a regimen of the nitric oxide donor molsidomine to improve the dystrophic phenotype of their muscles. The treatment indeed improved their muscle conditions, which was accompanied by a reversion (decrease) of the PC 34:1/PC 34:2 ratio to close to that in the healthy WT muscle. Their studies indicate that increased PC 34:1/PC 34:2 in *mdx* muscle occurs as part of the disease course, and in a reversible manner should the muscle pathology be improved. However, in other studies (Senoo et al., 2020) as well as the present study, it is clear that increased PC 34:1/PC 34:2 ratios variably manifest in affected *mdx* tissues. As PC 34:1 and PC 34:2 may harbor the bulk of the FA 18:1 and FA 18:2 chains, respectively, that are at altered levels in DMD muscles, understanding the basis for the discrepancies in reported PC 34:1/PC 34:2 ratios is critical if we are to effectively utilize the *mdx* model to elucidate the biological meaning of altered PC in DMD patients’ muscles. Accordingly, we have investigated possible causes of the discrepancies focused on three likely factors to impact PC compositions—muscle-type, age, and diet.

One source of variation in the *mdx*-phospholipid patterns was the muscle type. In adult healthy C576BL/6 mice, similar PC and PE patterns were observed among EDL, GAS, and TA, but the patterns in SOL differed more from the other muscles (Figures 1A,B). The expression patterns of genes related to the regulation of metabolic capacity and lipid metabolism also differed markedly in SOL from the other muscles (Figures 2A–F). The unique PC and PE patterns and metabolic gene expressions in SOL might reflect unique metabolic characteristics of this muscle related to its high content of slow-twitch and oxidative muscle fibers (Isaeva et al., 2005). In B10-WT and -*mdx* mice, the patterns and *mdx*-associated alterations of PC and PE species were also relatively similar in EDL and GAS but differed substantially in SOL (Figures 4A–C). In PC, the *mdx*-associated increases of PC 34:1 and PC 34:2 detected in adult EDL and GAS appeared diminished or nearly absent in SOL.

Age of muscle was another important factor influencing *mdx*-associated PC alterations. The PC alterations detected in mature adult *mdx*-muscles (12-week-old) were generally not detected in 2-week-old muscles, before disease onset, but were generally evident at age 6-weeks, when muscle regeneration was already underway (Figures 4A–C). In EDL (Figure 5A), the time course of change in levels of PC 34:1 and PC 34:2 differed, with PC 34:1 showing a rebound pattern in *mdx* tissue after disease onset suggesting reactivation of an early developmental program, while PC 34:2 increased in both *mdx* and WT tissues between ages 3–6-weeks, a period of rapid growth, suggesting that in mature adult *mdx* muscle (i.e., 12- or 18-weeks-old) high-*mdx* levels of PC 34:2 levels might reflect high nutritional demand of regenerating fibers, and therefore vary in its manifestation depending upon the nutritional supplies of fatty chains.

The time course of PC alterations observed in SOL differed from EDL and GAS in that the magnitude of the *mdx*-associated alterations were much diminished in the adult tissues and were maximally detected earlier, at age 3-weeks (Figure 5B). In *mdx* mice, SOL is reported to undergo a stage of early and severe degeneration, and it will be fascinating to examine in future studies whether the patterning of lipid alterations reflects or impacts the disease susceptibility for various muscle groups to *mdx* pathology, especially in relation to early or severe degeneration reported for muscles such as SOL (early) or diaphragm (severe) compared to other muscles (Carnwath and Shotton, 1987; Stedman et al., 1991).

Diet was a major factor to affect the *mdx*-specific PC alterations and is likely the major factor to influence the variability in different reports as to whether or not PC 34:1/PC 34:2 ratios were increased in affected *mdx* muscles. We found that PC 34:1 showed a consistent pattern of upregulation in affected *mdx* muscle regardless of diet, while PC 34:2 variably showed alterations in *mdx* muscle, depending on which diet mice were raised on. *Mdx* mice raised on CE-2 standard chow, a standard laboratory mouse diet in our institutions, showed increased PC 34:2 compared to WT mice, but this alteration was completely absent in mice raised on oleic- or linoleic-rich custom diets. These diets formulations resemble that of another common laboratory diet, AIN-93G. Unlike AIN-93G and the custom diets, CE-2 standard chow contains significant levels of DHA and EPA fish oils (Table 3). This or another variable between the CE-2 chow and the other diets may be a primary factor to cause the diet-dependent variations of patterns of PC 34:2 in *mdx* muscle, while the variation in oleic acid and linoleic acid levels between the two custom diets had less affect on the qualitative patterns of *mdx*-specific alterations (Figures 6A–C).

Although the current study highlights the PC alterations and the experimental variables that affect their detection in *mdx* muscle, it is limited in that it does not address the biological origin of the altered PC, nor how the PC alterations might affect disease onset, progression, or severity. More insight is also needed as to why *mdx*-specific PC alterations are less detectable in SOL than in EDL and GAS, and whether this is related to differences in the muscle pathologies and/or the fiber-type compositions. PC compositions vary between membranes of different organelles, and future studies must determine in which specific membranes of myofibers the PC alterations are manifested, such as plasma membranes, sarcoplasmic reticulum membranes, or mitochondrial membranes. This will also shed light on how the PC alterations might impact or reflect various pathological aspects of dystrophic muscle such as plasma membrane fragility, calcium mishandling, and mitochondrial dysfunction or deficit. Moreover, identification of the biological origin of the altered PC, including the lipid synthesizing or degrading molecules involved and impacts of nutrients, is required and will enable genetic- or pharmacological-based strategies to assess the disease impact or therapeutic potential of altered PC in dystrophic muscle.

In summary, our results indicate high PC 34:1 is a common characteristic of regenerating *mdx* muscle while alteration of PC 34:2 was variable and highly dependent on mouse diets. Other *mdx*-specific alterations in PC such as decreased PC 38:6 also occurred and might have significant biological impacts, as may additional *mdx*-associated alterations in less abundant species and other lipid classes not analyzed in our study. The present study focused on PC 34:1and PC 34:2 because these abundant PC species may be major repositories for the FA 18:1 and FA 18:2 chains in PC which are reported to be highly altered in the affected muscles of DMD patients. Further studies are required to determine the origin and biological significance of enhanced PC 34:1 and other lipid alterations in dystrophic muscle.

## Data Availability Statement

The original contributions presented in the study are included in the article/Supplementary Material, further inquiries can be directed to the corresponding author/s.

## Ethics Statement

The animal study was reviewed and approved by the Ethics Committee for animal experiments of the National Center for Global Health and Medicine, Tokyo, Japan.

## Author Contributions

WV designed the study, performed the experiments, analyzed the data, and wrote the manuscript. SM performed the experiments, analyzed the data, and interpreted the results. ST, FH, YK, and NI analyzed the data. JN and NM interpreted the results. YA wrote the manuscript. TS and HS designed and wrote the manuscript. All authors reviewed and agreed to the manuscript.

## Author Disclaimer

Department of Lipid Signaling, National Center for Global Health and Medicine is financially supported by Ono Pharmaceutical Co., Ltd. The Lipidomics Laboratory at the University of Tokyo is supported by Shimadzu Corporation.

## Conflict of Interest

The authors declare that the research was conducted in the absence of any commercial or financial relationships that could be construed as a potential conflict of interest.

## Publisher’s Note

All claims expressed in this article are solely those of the authors and do not necessarily represent those of their affiliated organizations, or those of the publisher, the editors and the reviewers. Any product that may be evaluated in this article, or claim that may be made by its manufacturer, is not guaranteed or endorsed by the publisher.
