# Supplementary material for: Lipidomic Analyses Reveal Specific Alterations of Phosphatidylcholine in Dystrophic Mdx Muscle
Source: Front Physiol. 2022 Jan 12;12:698166. doi: 10.3389/fphys.2021.698166 (PMC8791236; doi:10.3389/fphys.2021.698166)
Supplement: Supplementary file 1 [file Image_1.pdf]

Supplementary Figure 1

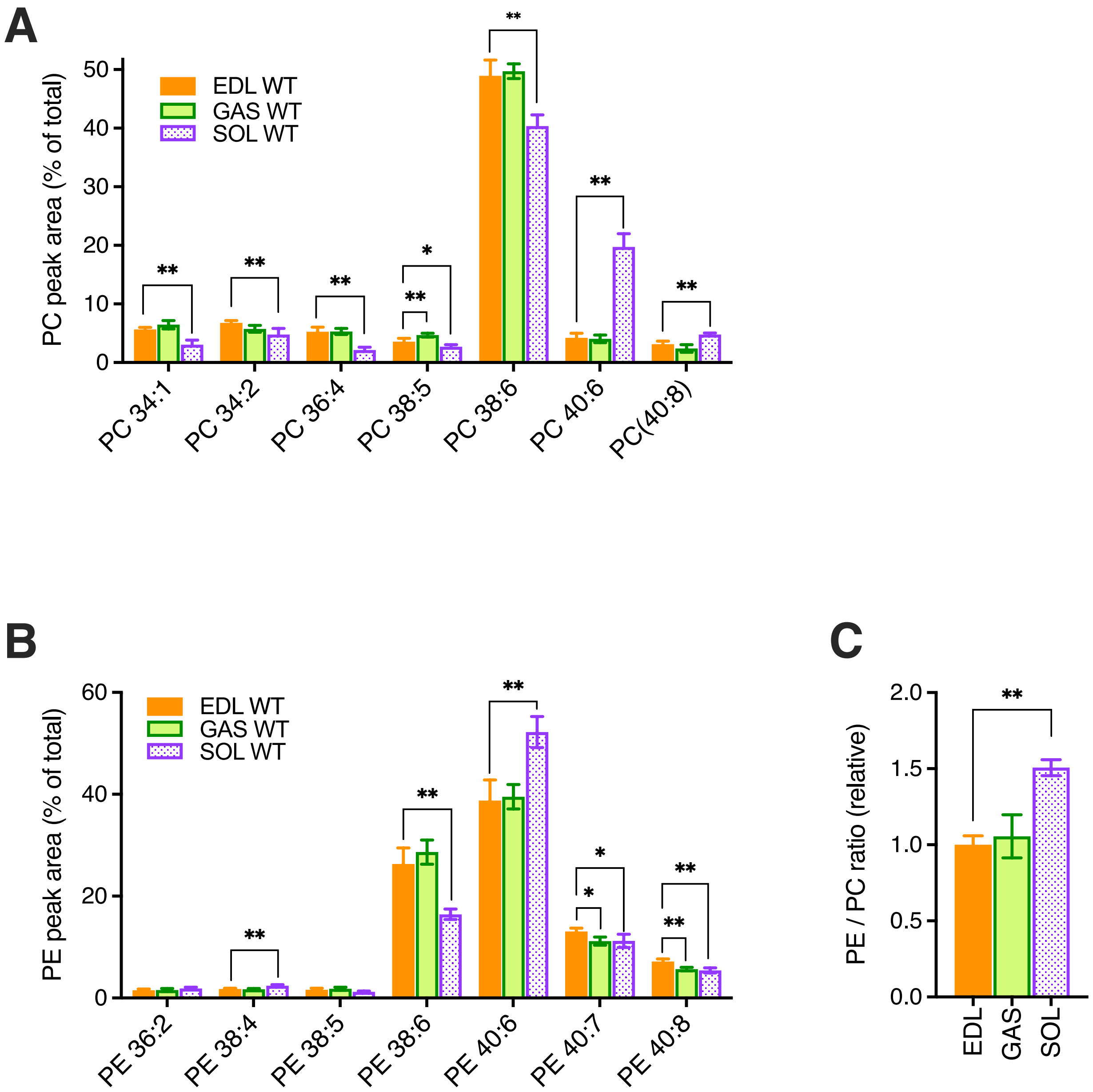

Supplementary Figure 1. PC and PE profiles in EDL, GAS, and SOL skeletal muscles of adult (18-week-old) WT-B10 mice raised on CE-2 standard chow. PC (A) and PE (B) were measured by LC-MS/MS. EDL was selected as a standard muscle to determine the statistical variations. (**C**) Relative ratios of total detected amounts of PE to PC were also determined (ratio in EDL = 1). PC and PE peak values are expressed as the percentage of total PC or PE signals, and means ± SD are plotted. Statistical significance of variations versus EDL is based on Dunnett's multiple comparison tests. *$p < 0.05$,**$p < 0.01$; n=5 mice/group.
