# Supplementary material for: Lipidomic Analyses Reveal Specific Alterations of Phosphatidylcholine in Dystrophic Mdx Muscle
Source: Front Physiol. 2022 Jan 12;12:698166. doi: 10.3389/fphys.2021.698166 (PMC8791236; doi:10.3389/fphys.2021.698166)
Supplement: Supplementary file 2 [file Image_2.pdf]

Supplementary Figure 2

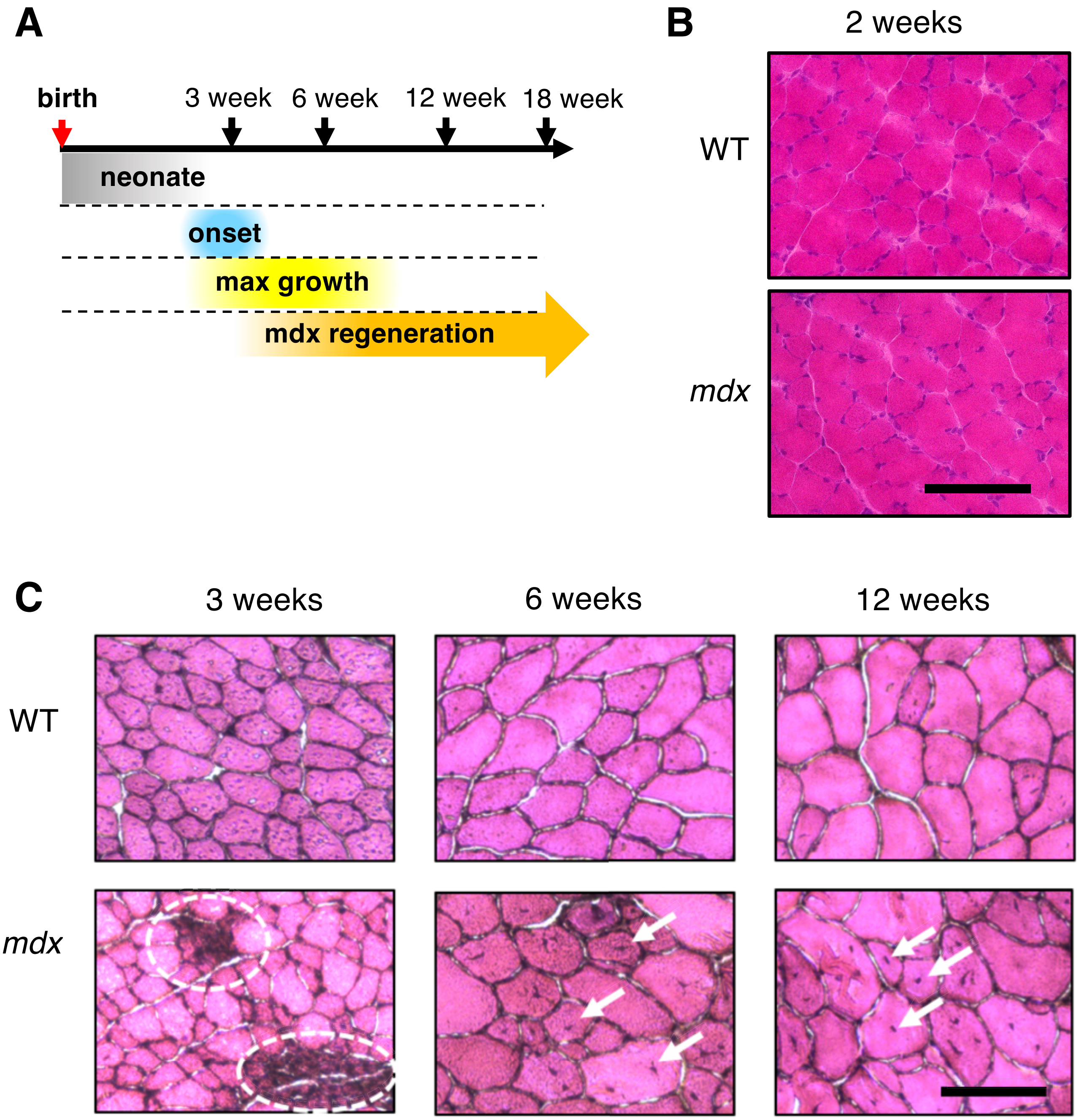

Supplementary Figure 2. Mouse growth and *mdx* disease progression. **(A):** General timeline of muscle pathology in *mdx* mice. **(B,C):** Hematoxylin and Eosin stained TA tissue sections of B10-WT and -*mdx* mice show examples of disease onset and regeneration in *mdx* muscle. **(B):** Two-week-old *mdx* TA muscle has not undergone degeneration and resembles healthy muscle. **(C):** Initial disease onset is marked by wave of degeneration/regeneration at ~3 weeks of age (outlined areas). In 6- and 12-week-old *mdx* muscle, central nuclei in myofibers (arrows) indicate active regeneration. Scale bars = 100 µm.
