# Supplementary material for: Lipidomic Analyses Reveal Specific Alterations of Phosphatidylcholine in Dystrophic Mdx Muscle
Source: Front Physiol. 2022 Jan 12;12:698166. doi: 10.3389/fphys.2021.698166 (PMC8791236; doi:10.3389/fphys.2021.698166)
Supplement: Supplementary file 3 [file Image_3.pdf]

Supplementary Figure 3

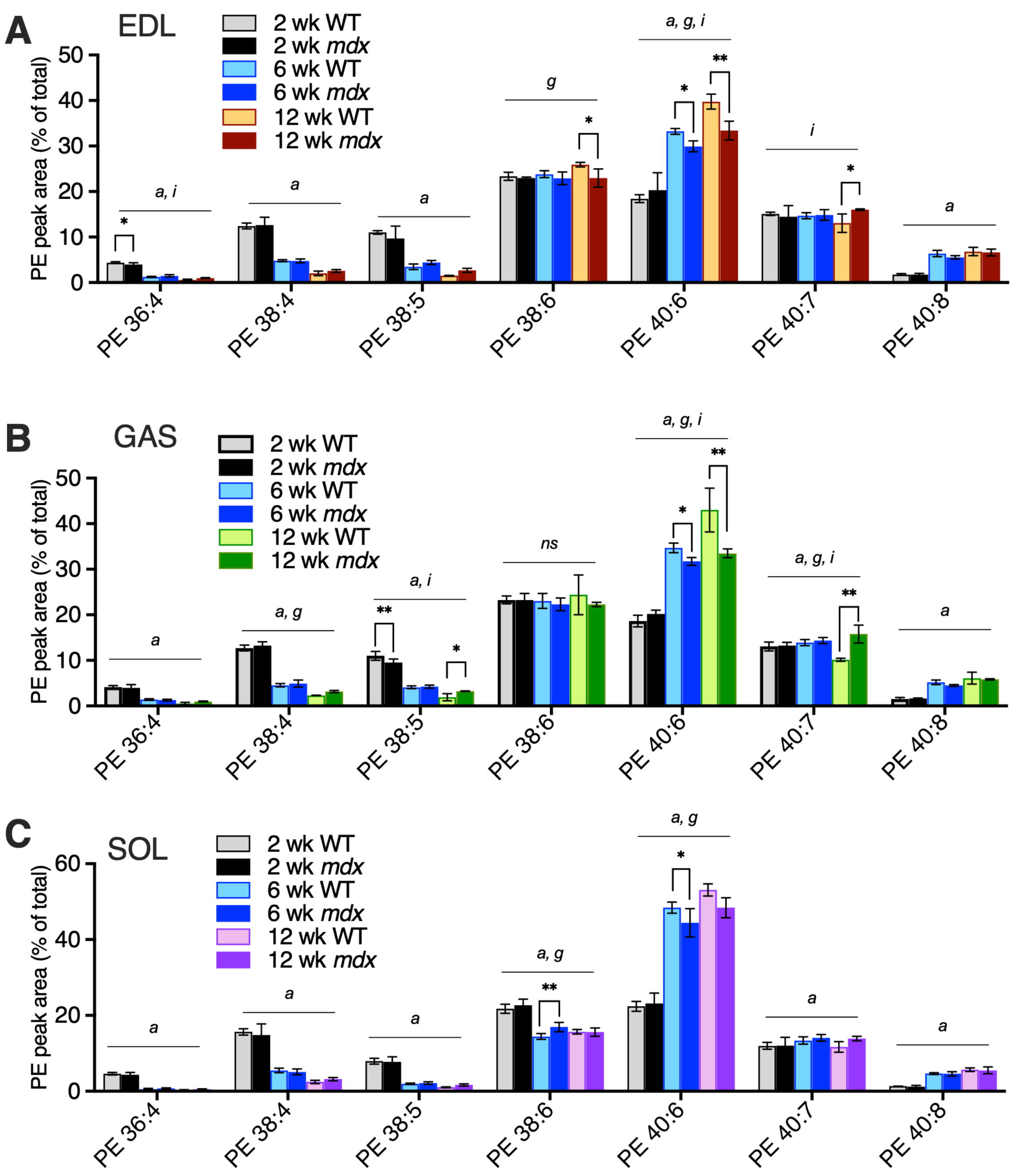

Supplementary Figure 3. PE alterations in EDL, GAS and SOL of B10-WT and -*mdx* mice of various ages (2-, 6-, and 12-week-old) raised on CE-2 standard chow. PE was measured in EDL (A), GAS (**B**), and SOL (**C**). PE peak values are expressed as the percentage of total signals, and means ± SD are plotted. Significant variation ($p < 0.05$) was determined by two-way ANOVA and factor effects for each peak are shown for age (*a*), mdx genotype (*g*), and interactive effects (*i*). Significant pair-wise differences between same-aged WT and *mdx* muscles were determined by Sidak's post tests; *$p < 0.05$,**$p < 0.01$. n=3-6 mice/group. *ns;* non-significant.
