# Supplementary material for: Lipidomic Analyses Reveal Specific Alterations of Phosphatidylcholine in Dystrophic Mdx Muscle
Source: Front Physiol. 2022 Jan 12;12:698166. doi: 10.3389/fphys.2021.698166 (PMC8791236; doi:10.3389/fphys.2021.698166)
Supplement: Supplementary file 4 [file Image_4.pdf]

Supplementary Figure 4

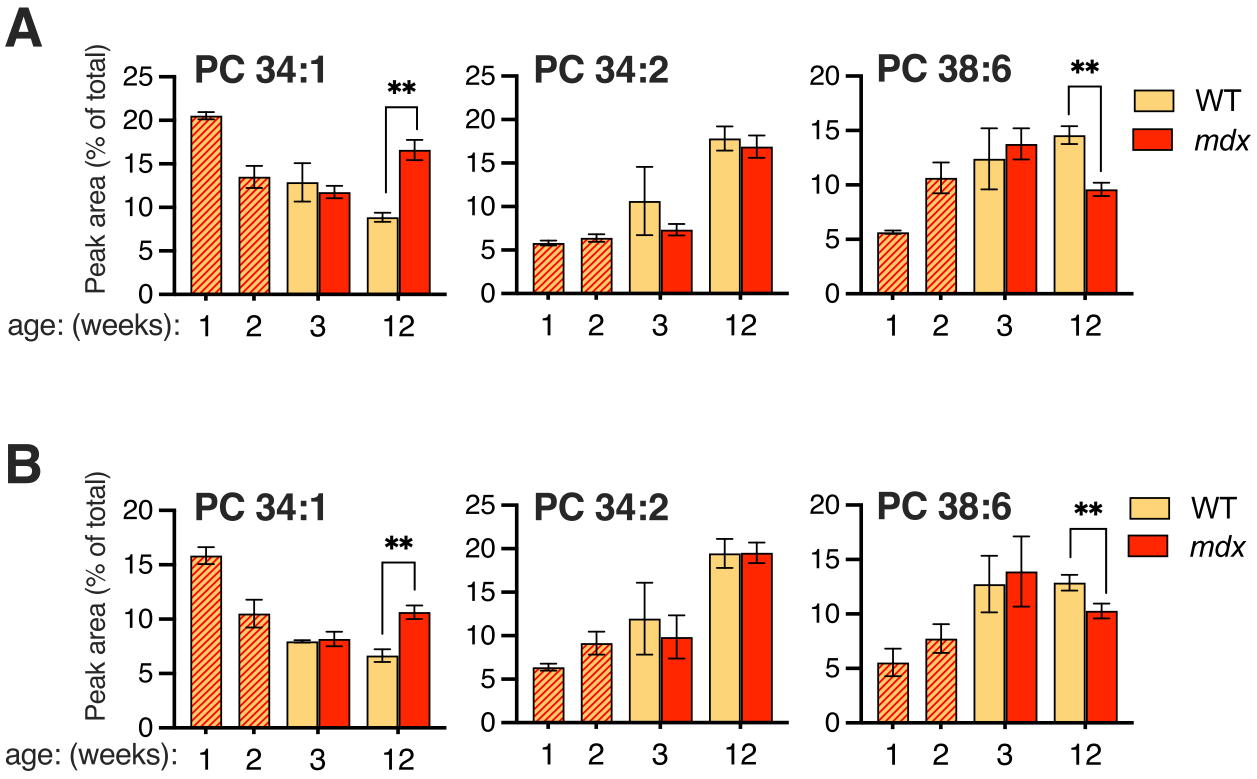

Supplementary Figure 4. Time course of PC 34:1, PC 34:2, and PC 38:6 alterations in EDL muscles of 1- to 12-week-old B10-WT and -mdx mice fed custom diets. Mice were raised on custom diets rich in FA 18:1/oleic acid (oleic) (**A**) or FA 18:2 /linoleic acid (linoleic) (**B**). Similar patterns of alterations were seen in both custom diets, with the mdx-associated alterations of high PC 34:1 and low PC 38:6 evident at age 12-weeks, but alterations of PC 34:2 were not detected. PC peak values are expressed as the percentage of total PC signals, and means ± SD are plotted. 1- and 2-week-old groups (hatched bars) contain both WT and mdx, male and female, pups, and are shown for reference. Includes identical data (12-week time point) shown in Figure 6. Significance is based on unpaired t-tests. *p < 0.05,**p < 0.01; n=3-8 mice/group.
